# Transcriptome Analysis of Jujube (*Ziziphus jujuba* Mill.) Response to Heat Stress

**DOI:** 10.1155/2021/3442277

**Published:** 2021-12-02

**Authors:** Lei Yang, Juan Jin, Dingyu Fan, Qing Hao, Jianxin Niu

**Affiliations:** ^1^Department of Horticulture, College of Agriculture, Shihezi University, Shihezi, 832003 Xinjiang, China; ^2^Xinjiang Production and Construction Corps Key Laboratory of Special Fruits and Vegetables Cultivation Physiology and Germplasm Resources Utilization, Shihezi, 832003 Xinjiang, China; ^3^Institute of Horticulture Crops, Xinjiang Academy of Agricultural Sciences, Urumqi, 830091 Xinjiang, China; ^4^Scientific Observing and Experimental Station of Pomology (Xinjiang), Urumqi, 830091 Xinjiang, China

## Abstract

Heat stress (HS) is a common stress influencing the growth and reproduction of plant species. Jujube (*Ziziphus jujuba* Mill.) is an economically important tree with strong abiotic stress resistance, but the molecular mechanism of its response to HS remains elusive. In this study, we subjected seedlings of *Z. jujuba* cultivar “Hqing1-HR” to HS (45°C) for 0, 1, 3, 5, and 7 days, respectively, and collected the leaf samples (HR0, HR1, HR3, HR5, and HR7) accordingly. Fifteen cDNA libraries from leaves were constructed for transcriptomics assays. RNA sequencing and transcriptomics identified 1,642, 4,080, 5,160, and 2,119 differentially expressed genes (DEGs) in comparisons of HR1 vs. HR0, HR3 vs. HR0, HR5 vs. HR0, and HR7 vs. HR0, respectively. Gene ontology analyses of the DEGs from these comparisons revealed enrichment in a series of biological processes involved in stress responses, photosynthesis, and metabolism, suggesting that lowering or upregulating expression of these genes might play important roles in the response to HS. This study contributed to our understanding of the molecular mechanism of jujube response to HS and will be beneficial for developing jujube cultivars with improved heat resistance.

## 1. Introduction

Abiotic stresses, such as heat, drought, salinity, and cold, are major environmental constraints to crop production and food security all over the world [1, 2]. In particular, with extreme weather and global warming, heat stress (HS) has received increasing concern and interest [3]. Although increasing temperatures are beneficial for crop production in some cooler regions of the world, their overall impact on global food security is negative [4]. HS harms cellular homeostasis and causes leaf etiolation, severe retardation in growth and development, increased risk of disease, and even death [5]. Temperature increases reduces global yields of major crops, such as wheat, rice, maize, and soybean [6], in addition to horticultural crops such as grapevine, almonds, apples, oranges, and avocados [7].

Jujube (*Ziziphus jujuba* Mill.) belongs to the Rhamnaceae family in the Rosales order [8] and is one of the oldest cultivated horticultural crops with a long domestication history [9]. Jujube fruit is rich in vitamin C, phenolics, flavonoids, triterpenic acids, and polysaccharides and is widely consumed as a food or food additive [10]. It is now a major dry fruit crop with a cultivation area of 2 million ha, the main source of income for 20 million farmers, and a traditional herbal medicine for more than one billion people in Asia [11]. Jujube is well adapted to various biotic and abiotic stresses, especially drought and salinity, and is considered an ideal cash crop for arid and semiarid areas where common fruits and grain/oil crops do not grow well [8].

Xinjiang province of China is the core area of the arid region in Central Asia, which is one of the most arid regions in the world [12]. Jujubes are among the main agroeconomically important crops in Xinjiang, and those from this region have good quality and the highest production worldwide [13]. However, Xinjiang is particularly vulnerable to climate change and has experienced significant climate warming in the past 40 years [14]. In recent years, HS has dramatically and repeatedly affected the production of quality jujube fruits in Xinjiang. Therefore, identifying and breeding heat-tolerant jujube cultivars might be a feasible and important strategy for protecting the production and quality of jujube fruits.

Turpan, one of the northeastern cities of in Xinjiang province of China, has a unique temperate continental arid desert climate, with bright sunshine, high temperatures, and large day-night differences in temperature [15]. We found a jujube cultivar (“Hqing1-HR”) in an orchard of Turpan by chance [16] and bred it in our laboratory successfully. To investigate the transcriptomic change in “Hqing1-HR” response to HS, we subjected its seedlings to 45°C stress. At 0, 1, 3, 5, and 7 days after HS-treatment, we assessed phenotypic and physiological features and collected samples for RNA sequencing experiments. This study may provide new insight into transcriptional alterations in heat-resistant jujube cultivars responding to HS.

## 2. Materials and Methods

### 2.1. Plant Materials, Heat Treatment, and Sample Collection

Green cuttings of “Hqing1-HR” were collected from the jujube orchard of Turpan in Xinjiang, China. The green cuttings were grown under greenhouse conditions with an automatic spray system (20~35°C with 90% humidity). When the cuttings had 7~9 true leaves, a total of 80 plants were transferred to a controlled growth chamber with a light/dark regime of 14/10 h at 30/20°C, 80% relative humidity, and light intensity of 300 *μ*mol m^−2^ s^−1^ of photosynthetically active radiation.

Seedlings with 14 true leaves were cultured in the same chamber under the same conditions except with the temperature at 45°C. After 0 (control), 1, 3, 5, and 7 days of heat treatment, the 10th true leaves counting from bottom to top were collected from three different samples as biological repetitions. Leaf samples were immersed in liquid nitrogen and stored at -80°C for transcriptome sequencing.

### 2.2. Phenotypic Determination of Jujube Leaves

Samples from the same part of each leaf were rinsed and fixed with FAA solution (70% ethanol) at 4°C. Leaves were freeze-dried after dehydration using an alcohol gradient series to the critical drying point and then stuck to the table using conductive tape. Samples were coated with a Pt film using an ion sputtering instrument (Hitachi E-1045), and a SUPRA 55VP scanning electron microscope (Zeiss, German) was used for observation of leaves at an accelerating voltage of 2.00 kV.

### 2.3. RNA Extraction, cDNA Library Construction, and Illumina Sequencing

Total RNAs were extracted from 15 samples representing three biological replicates of “Hqing1-HR” at five treatment stages (0, 1, 3, 5, and 7 days of HS) using an RNAprep Pure Plant Kit (Tiangen, Beijing, China) according to the instructions of the manufacturer. Extracted RNA was treated with DNase I (Promega, Madison, WI, USA) to remove DNA. RNA quality and quantity were assessed using a Nanodrop 2000 Spectrophotometer (Thermo Fisher Scientific, Wilmington, DE, USA) and an Agilent Bioanalyzer 2100 System (Agilent Technologies, Santa Clara, CA, USA), respectively. The integrity of RNA was confirmed by 1% (*w*/*v*) agarose gel electrophoresis.

RNA samples containing equal amounts of RNA were pooled from three independent individuals and then used for library preparation and sequencing. The resulting libraries were sequenced using an Illumina HiSeq X-ten platform with paired-end 150 bp reads.

### 2.4. RNA-seq Read Processing and Assembly

Raw reads were generated by the Illumina HiSeq X-ten genome analyzer and were analyzed using Fast Q to assess the base quality. Reads were cleaned by removing adaptor sequences, low-quality sequences including empty reads, and sequences containing <10% bases with a Phred quality score < 20. The transcriptome was assembled using StingTie V1.3.1 [17], and the remaining cleaned reads were mapped to the jujube reference genome sequences [11] using HISAT2 [18] with default settings.

### 2.5. Bioinformatic Analysis

FPKM (fragments per kilobase per million mapped reads) was used to evaluate the expression level of genes. The software edge R [19] was used to measure the FPKM values and identify differentially expressed genes (DEGs). Genes with RPKM < 0.1 in every sample were removed before analysis. DEGs were determined on the basis of fold change (FC) (FC ≥ 2 or ≤ 0.5) and false discovery rate (FDR) (FDR < 0.01).

To predict gene function and calculate the distribution frequency of functional categories, Gene Ontology (GO) analyses were employed using DAVID bioinformatics resources [20]. Venn diagrams were generated using a tool available online (http://bioinfogp.cnb.csic.es/tools/venny/).

### 2.6. Validation of RNA-seq Using Quantitative Reverse-Transcription PCR (qRT-PCR)

To verify the validity of the RNA-seq data, qRT-PCR experiments were performed for randomly selected DEGs. Primers are presented in Table 1. The same RNA samples used for RNA-seq were used for qRT-PCR. In each pooled sample, l *μ*g of RNA was reverse transcribed using a Prime Script™ RT Reagent Kit (Takara, Dalian, China) according to the instructions of manufacturer. The qPCR was performed on a Bio-Rad S1000 with Bestar SYBR Green RT-PCR Master Mix (DBI Bioscience, Shanghai, China). PCR conditions were as follows: denaturing at 95°C for 8 min, 38 cycles of denaturing at 95°C for 15 s, and annealing and extension at 60°C for 1 min. Relative gene expression was calculated using the Livak and Schmittgen 2^−*ΔΔ*Ct^ method [21], normalized with the reference gene *ZjH3* of jujube. PCR amplifications were performed in triplicate for each sample.

### 2.7. Statistical Analysis

All values of all data are presented as mean ± standard deviation (SD). To determine the significance of differences between means, Student's *t*-test (paired) was implemented, and a value *P* < 0.05 was considered statistically significant.

## 3. Results

### 3.1. Phenotype of Jujube Seedlings Post HS

**Figure 1:**
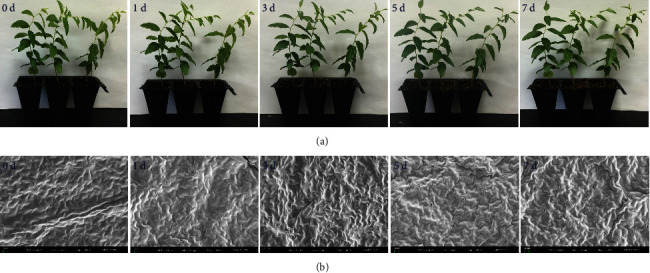
Effects of high temperature stress on the phenotypic of jujube. Macroscopic phenotypes (a) and stomatal morphology (b) of heat-tolerant seedlings under different durations of high-temperature stress.

To obtain an overview of the heat-tolerance phenotype of “Hqing1-HR,” seedlings with 14 true leaves were subjected to treatment with HS (45°C). At 0 (control) 1, 3, 5, and 7 days after the treatment, none of the seedlings displayed withered leaves (Figure 1(a)), suggesting this jujube cultivar might be of heat tolerant.

Stoma are important channels for gas and water exchange between plants and the atmosphere and can make adaptive adjustments under various stress conditions. We assessed the stomatal density and stomatal opening rate of leaves from each group. Stomatal density and stomatal opening rate were significantly increased post heat treatment, and they showed a trend of first increasing and then decreasing with the extension of heat treatment (Figure 1(b), Table 2). This suggested that the “Hqing1-HR” could reduce the damage by passively changing stomatal density and opening rate.

### 3.2. RNA-seq Data Summary

We prepared the seedling samples (HR0, HR1, HR3, HR5, and HR7) for the “Hqing1-HR” cultivar on days 0, 1, 3, 5, and 7 post heat treatment at 45°C, respectively, and constructed 15 cDNA libraries (HR0-a, HR0-b, and HR0-c; HR1-a, HR1-b, and HR1-c; HR3-a, HR3-b, and HR3-c; HR5-a, HR5-b, and HR5-c; and HR7-a, HR7-b, and HR7-c) for RNA-seq, representing three biological replicates at each time point.

Through the Illumina HiSeq X-ten platform, we generated over 0.402 billion paired-end reads, corresponding to an average of 26.8 million sequence reads per sample. Using HISAT2 [18] with default settings, approximately 68.9% clean reads were mapped to the jujube reference genome [11].

**Figure 2:**
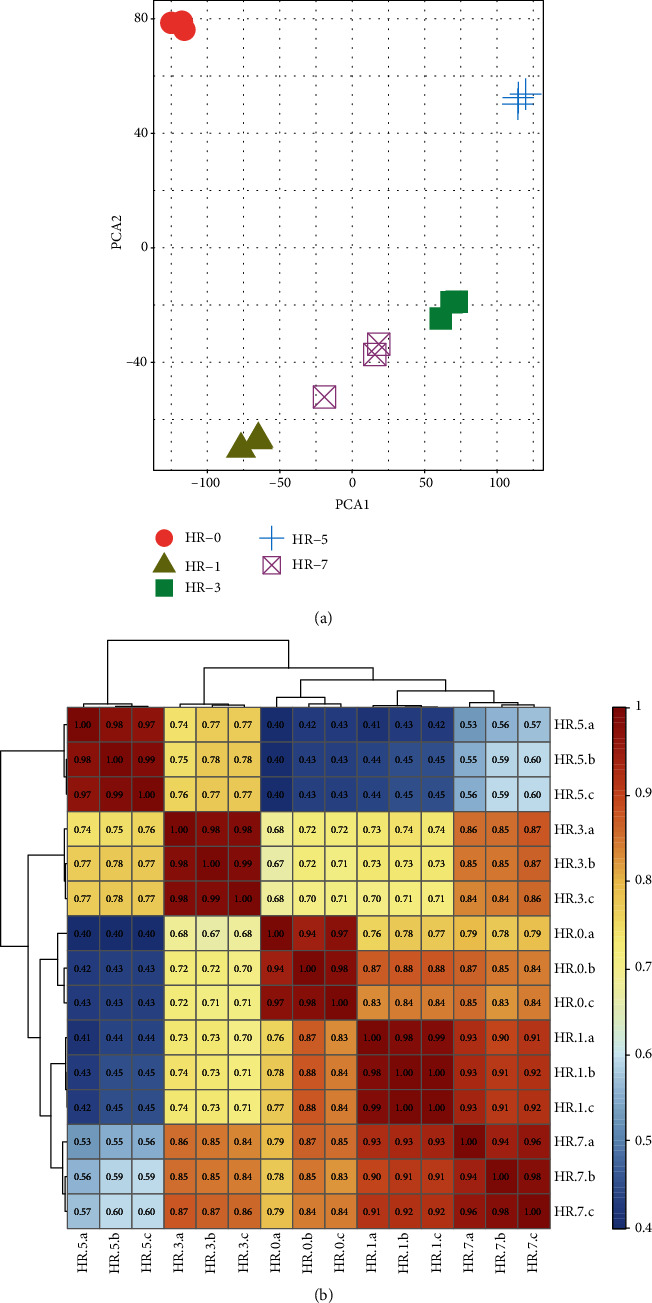
Discrete expression patterns of mRNAs. (a) Principal component analysis (PCA) of 15 distinct samples across five time points based on normalized mRNAs expression levels. Samples are grouped by brain region, and the ellipse for each group indicates confidence. (b) Heatmap of correlations for 15 samples based on the mRNAs expression levels.

To understand the spatiotemporal expression patterns of all samples, we performed principal component analysis (PCA). The three samples collected at each time point formed independent clusters (Figure 2(a)). Moreover, Pearson's correlation analysis for all pairs of RNA-seq samples was performed, demonstrating similar results (Figure 2(b)) and indicating that gene expression in the three replications of every sample was homogeneous (Figure 2(a)). This suggested that the replicated samples produced data acceptable for further analyses.

### 3.3. Exploration of Differentially Expressed Genes (DEGs)

**Figure 3:**
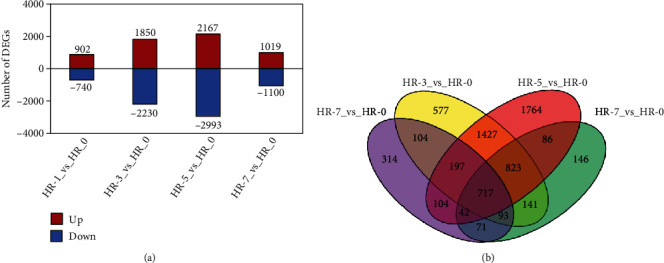
Exploration of differentially expressed genes (DEGs) (FC ≥ 2 or ≤ 0.5, FDR ≤ 0.05). (a) Number of up- and downregulated DEGs in four comparisons. (b) Venn diagrams of DEGs from HR7 vs. HR0, HR5 vs. HR0, HR3 vs. HR0, and HR1 vs. HR0 comparisons.

Using the software edge R [19], we detected 1,642, 4,080, 5,160, and 2,119 DEGs (FC ≥ 2 or ≤ 0.5, FDR ≤ 0.01) in the comparisons of HR1 vs. HR0, HR3 vs. HR0, HR5 vs. HR0, and HR7 vs. HR0, respectively, with the highest and lowest FC being 2^14.08^ and 2^−14.12^, respectively (Figure 3(a), Tables S1-S4). This indicated that heat stress leads to comprehensive transcriptome changes in cells of the jujube leaves.

In HR1 vs. HR0, we identified 902 upregulated genes and 740 downregulated genes. There were 1,850 upregulated and 2,230 downregulated genes in HR3 vs. HR0. In HR5 vs. HR0, 2,167 upregulated and 2,993 downregulated genes were discovered. Meanwhile, 1,019 upregulated and 1,100 downregulated genes were identified in HR7 vs. HR0. The numbers of the upregulated and downregulated DEGs were similar in each comparison, indicating that heat stress promoted and inhibited the transcription of numerous genes. Moreover, there were 717 common DEGs among the four comparisons (Figure 3(b)).

### 3.4. Molecular Response to Heat Stress

To identify the pathways in which the DEGs were mainly involved, we conducted Gene Ontology (GO) enrichment analysis. This revealed 36, 58, 65, and 37 GO terms in HR1 vs. HR0, HR3 vs. HR0, HR5 vs. HR0, and HR7 vs. HR0, respectively (*P* < 0.01).

**Figure 4:**
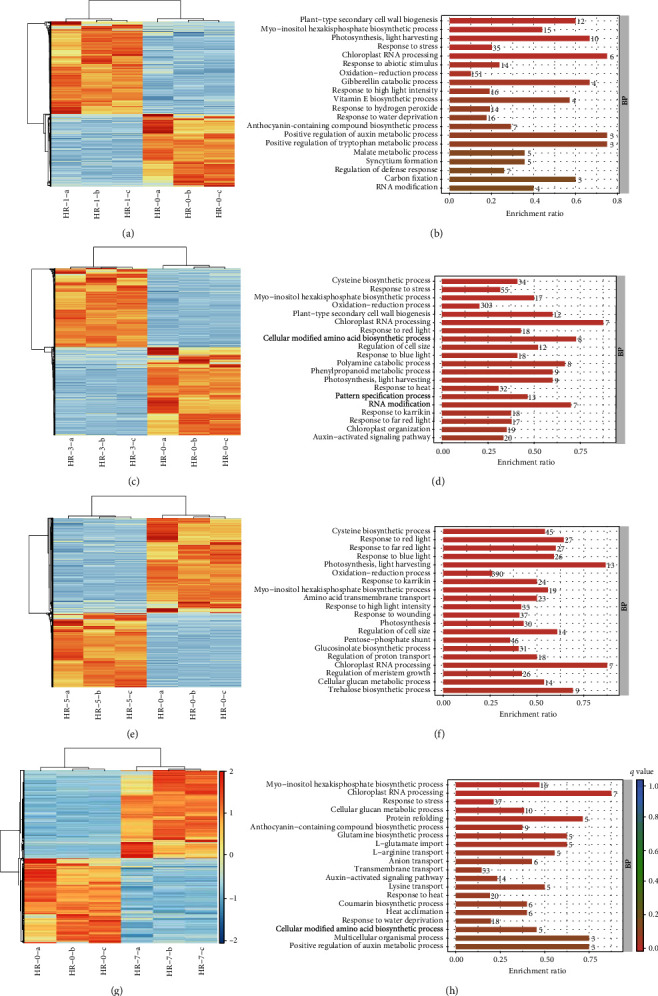
The GO analysis of differentially expressed genes (DEGs) from HR-1 vs. HR-0, HR-3 vs. HR-0, HR-5 vs. HR-0, and HR-7 vs. HR-0. (a, c, e, and g) Heatmaps for DEGs from HR-1 vs. HR-0, HR-3 vs. HR-0, HR-5 vs. HR-0, and HR-7 vs. HR-0, respectively. Heatmaps were generated from the hierarchical analysis of genes and samples. (b, d, f, and h) GO analyses of DEGs from HR-1 vs. HR-0, HR-3 vs. HR-0, HR-5 vs. HR-0, and HR-7 vs. HR-0, respectively. Only the top 20 terms are listed here.

Although leaves of none of the seedlings become withered under high temperature, multiple DEGs were associated with “response to stress” (GO: 0006950) and “response to heat” (GO: 0009408) terms in all four comparisons (Figure 4 and Tables S5-S8), indicating that “Hqing1-HR” might be sensitive to HS under normal conditions but establish a new steady-state balance of biological processes enabling the organism to function and survive well at higher temperatures (45°C). Analysis of DEGs enriched in “response to stress” and “response to heat” terms indicated that the expression levels of multiple DEGs associated with the response to HS were clearly upregulated after HS. Previous study indicated that cells can activate an ancient signaling pathway leading to the transient expression of HSPs in response to heat stress [22]. Indeed, it was found that multiple DEGs encode the heat shock proteins (HSPs), including HSP17 (gene12298), HSP18 (gene3931 and gene3933), HSP21 (gene2239), HSP 22 (gene7584), HSP 23 (gene6955), HSP26 (gene22046), HSP70 (gene2890 and gene4042), HSP83 (gene21597), HSP90 (gene9467), HSF30 (gene12331), and HSC-2 (gene22447) in the current study.

### 3.5. Photosynthesis Is Affected by Heat Stress

Photosynthesis occurs in chloroplasts and is sensitive to high temperatures. We identified “chloroplast organization” (GO: 0009658) and “chloroplast RNA processing” (GO: 0031425) terms in the HR1 vs. HR0, HR3 vs. HR0, HR5 vs. HR0, and HR7 vs. HR0 comparisons, implying that the normal physiology of chloroplasts and photosynthesis is affected by HS. The “photosynthesis, light harvesting” (GO: 0009765) and “photosynthesis” (GO: 0015979) terms were found in HR1 vs. HR0, HR3 vs. HR0, and HR5 vs. HR0 (Figure 4 and Tables S5-S7). Despite these terms not being enriched in HR7 vs. HR0, some DEGs belonging to the two terms were identified.

To explore how HS affected the normal physiology of chloroplasts and photosynthesis, we analyzed the DEGs associated with “chloroplast organization” (GO: 0009399), “chloroplast RNA processing” (GO: 0031425), “photosynthesis, light harvesting” (GO: 0009765), and “photosynthesis” (GO: 0015979) terms. To our surprise, most of the DEGs enriched in these four terms above were upregulated by HS. This suggested that HS might not disrupt the physiology of chloroplasts or photosynthesis and instead might promote the photosynthesis in “Hqing1-HR.”

### 3.6. Metabolisms Is Affected by Heat Stress

HS always affects the global metabolism of plants. “Myo-inositol hexakisphosphate biosynthetic process” (GO: 0010264) was the only common term associated with metabolism identified in HR1 vs. HR0, HR3 vs. HR0, HR5 vs. HR0, and HR7 vs. HR0. However, we identified multiple specific terms associated with metabolism in the four comparisons (Figure 4 and Tables S5-S8).

In HR1 vs. HR0, “malate metabolic process” (GO: 0006108), “anthocyanin-containing compound biosynthetic process” (GO: 0009718), “plastoquinone biosynthetic process” (GO: 0010236), “negative regulation of nucleotide metabolic process” (GO: 0045980), “vitamin E biosynthetic process” (GO: 0010189), and “monocarboxylic acid biosynthetic process” (GO: 0072330) terms were identified.

In HR3 vs. HR0, we found “cellular modified amino acid biosynthetic process” (GO: 0042398), “phenylpropanoid metabolic process” (GO: 0009698), “glutamine biosynthetic process” (GO: 0006542), “nucleotide-sugar metabolic process” (nucleotide-sugar metabolic process), “polyamine catabolic process” (GO: 0006598), “anthocyanin-containing compound biosynthetic process” (GO: 0009718), “cutin biosynthetic process” (GO: 0010143), “positive regulation of flavonoid biosynthetic process” (GO:0009963), “glutathione catabolic process” (GO:0006751), “wax biosynthetic process” (GO:0010025), “glutamate biosynthetic process” (GO: 0006537), “glycine betaine biosynthetic process” (GO:0031456), “positive regulation of auxin metabolic process” (GO:0090355), and “positive regulation of tryptophan metabolic process” (GO:0090358) terms.

In HR5 vs. HR0, “cysteine biosynthetic process” (GO: 0019344), “positive regulation of catalytic activity” (GO: 0043085), “glucosinolate biosynthetic process” (GO: 0019761), “glycogen biosynthetic process” (GO: 0005978), “cellular glucan metabolic process” (GO: 0006073), “hydrogen peroxide catabolic process” (GO: 0042744), “trehalose biosynthetic process” (GO: 0005992), “tryptophan catabolic process” (GO: 0006569), “long-chain fatty acid metabolic process” (GO: 0001676), “indoleacetic acid biosynthetic process” (GO:0009684), “sucrose metabolic process” (GO:0005985), “glutathione catabolic process” (GO:0006751), “phenylpropanoid biosynthetic process” (GO:0009699), “malate metabolic process” (GO:0006108), “glutamine biosynthetic process” (GO:0006542), “starch biosynthetic process” (GO:0019252), and “cellular carbohydrate metabolic process” (GO:0044262) terms were identified.

In HR7 vs. HR0, we found “anthocyanin-containing compound biosynthetic process” (GO: 0009718), “glutamine biosynthetic process” (GO: 0006542), “cellular glucan metabolic process” (GO: 0006073), “positive regulation of catalytic activity” (GO: 0043085), “malate metabolic process” (GO: 0006108), “coumarin biosynthetic process”, “plastoquinone biosynthetic process” (GO: 0010236), “cellular modified amino acid biosynthetic process” (GO: 0042398), “polyamine catabolic process” (GO:0006598), and “wax biosynthetic process” (GO:0010025) terms.

### 3.7. Validation of RNA-seq by qRT-PCR

**Figure 5:**
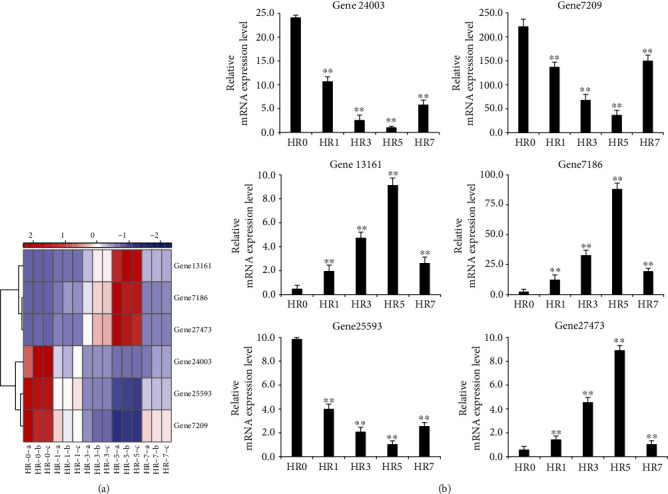
Quantitative reverse-transcription PCR (qRT-PCR) validation of differentially expressed genes (DEGs) identified from RNA-seq. (a) Heatmap was constructed for DEGs on the basis of log_2_ FPKM values ranging from blue (low expression) to red (high expression) at each time point. FPKM (fragments per kilobase per million mapped reads) was used to calculate expression levels of genes. (b) mRNA expression levels of the DEGs above were validated by qRT-PCR, normalized to the *H3* gene. Data represent the mean values ± SD. ^∗^*P* < 0.05; ^∗∗^*P* < 0.01, calculated using Student's *t*-test.

To verify the reliability of our transcriptome data, six DEGs were randomly selected for expression analysis using qRT-PCR experiments (Figure 5). The expression patterns shown by the qRT-PCR results (Figure 5(b)) were consistent with the RNA-seq results (Figure 5(a)), with PCCs higher than 0.9.

## 4. Discussion

The global air temperature is predicted to rise by 0.2°C per decade, which will lead to temperatures 1.8-4.0°C higher than the current level by 2100 [23]. HS is therefore becoming an agricultural problem in many areas in the world. HS generally impairs photosynthetic activity and reduces water content caused by heat and has negative effects on cell division and growth of crops. Thus, HS is a major environmental factor limiting crop productivity, and identifying and breeding the heat-tolerant cultivars of crops are a valuable way to protect food production and ensure crop safety [24, 25]. For example, the heat-tolerant cultivars have been identified in some major crops, including rice [26], maize [27], and wheat [28]; however, heat-tolerant cultivars of horticultural crops are seldom reported. In the current study, we found a putative heat-tolerant jujube (*Ziziphus jujuba* Mill.) cultivar (“Hqing1-HR”) that can survive under serious HS (45°C). To our knowledge, this is the first report of a heat-tolerant cultivar of jujube. “Hqing1-HR” could be used to breed more heat-tolerant lines in the future.

Under high-temperature conditions, plants exhibit short-term avoidance or acclimation mechanisms such as transpirational cooling and stomatal closure [29]. We observed no macroscopic phenotypic differences, such as wilting, leaf curl, or yellowing, in “Hqing1-HR” jujube seedlings under different durations of high-temperature stress (Figure 1). However, scanning electron microscopy of leaves revealed that stomatal density and opening rate of leaves were significantly affected by high-temperature stress, showing a trend of rapid increase and then slow decrease with the extension of high temperature stress duration. Similar results have been reported in annual plants, such as soybean [30] and rice [31]. Stomatal development is very sensitive to fluctuations in environmental conditions such as temperature, osmotic stress, and carbon dioxide concentration [32]. HS affects the expression of *HSP90* [33, 34], *MUTE* [35], *SBH1* [36], *AGL16* [37], and other genes that are considered regulators of stomatal differentiation by orchestrating the transcriptional network controlling symmetric divisions. In the current study, multiple upregulated DEGs have the capacities to produce HSPs, including HSP17, HSP18, HSP21, HSP 22, HSP 23, HSP26, HSP70, HSP83, HSP90, HSF30, and HSC-2, suggesting HSPs might be molecular chaperones which prevent the formation of nonspecific and harmful protein aggregates and assist proteins in the acquisition of their native structures.

The physiological effects of HS on plants have been extensively reported, but our understanding of the underlying molecular mechanisms remains limited. Expression levels of multiple genes are affected by HS; thus, RNA-seq analysis, which provides precise information on the transcriptomic changes occurring in response to abiotic stress, including HS, is a suitable method for elucidating these mechanisms. For example, transcriptome profiling of rice [26], barley [38], maize [39, 40], *Brachypodium distachyon* [41], and *Vitis vinifera* (grape) [7, 42] in response to HS has generated useful clues associated with the molecular mechanism of the response to HS. In jujube species, RNA-seq experiments have also been performed to explore the transcriptomic changes that occur in response to abiotic stress, including drought stress [43] and alkalinity stress [44]. In the current study, we performed transcriptomic analysis for the jujube response to HS using RNA-seq experiments. These indicated that HS changes global expression levels of multiple genes, and we found 1,642, 4,080, 5,160, and 2,119 DEGs in HR1 vs. HR0, HR3 vs. HR0, HR5 vs. HR0, and HR7 vs. HR0 comparisons, respectively. Moreover, functional analyses indicated that a considerable number of DEGs were enriched in terms associated with photosynthesis metabolism, suggesting that “Hqing1-HR” might be tolerant to HS by upregulating or lowering the expression levels of these genes.

## 5. Conclusions

In this study, high temperature did not damage to the macroscopic phenotype of “Hqing1-HR.” However, stomatal density and opening rate were significantly affected by high-temperature stress. We conducted the transcriptome analysis of leaves and characterization of transcripts related to high-temperature stress during the seedling stage in jujube using a next-generation sequencing approach. A total of 6,606 DEGs were identified in “Hqing1-HR” under heat stress compared with the control treatment, and 1,642, 4,080, 5,160, and 2,119 DEGs were found in HR1 vs. HR0, HR3 vs. HR0, HR5 vs. HR0, and HR7 vs. HR0, respectively. The GO enrichment analysis showed that a series of biological processes related to stress response, photosynthesis, and metabolism were enriched during high-temperature stress, suggesting that down- or upregulation of genes in these processes may play an important role in the response to HS. These results contributed to our understanding of the molecular mechanism of jujube responses to high-temperature stress.

## Figures and Tables

**Table 1:** The genes and primers used for qRT-PCR experiments.

Gene	Forward primer (5′-3′)	Reverse primer (5′-3′)
*gene24003*	TGGCTGCTTCAGAGGTTTCG	CTATCTCACCAGGAACTCCCATT
*gene7209*	CGGCCCGATAACTTCGTCTT	CAGTTCTCAGCCTCCTTCCTCA
*gene13161*	CGGTGGCAGCAGTATCGTT	GTTCAGGTGGTCCCGCAAT
*gene7186*	GCAGCATCGGCGAATACAAA	CTTGGAAGCGACGGCATT
*gene25593*	AAAGGCTAATATGCTCAAGAGTGTG	CATAACGGAGCGTGGAGTGC
*Gene27473*	CTATTGCTGCCACCGCTCTT	GAAAGCCAAACAATGAATCACC
*ZjH3*	GAAGCAACTGGCAACTAAGGC	CGAACAGACCGACCAAGTAAGC

**Table 2:** Effect of high temperature stress on stomatal density and stomatal opening rate of seedlings.

	Heat treatment time (d)				
	0	1	3	5	7
Stomatal density (number/figure)	15.44c	15.93bc	19.19a	19.75a	18.75b
Stomatal opening rate (%)	38.49c	43.50c	72.31a	59.81b	58.00b

Note: stomatal density is represented by the number of pores in the picture. Stomatal opening rate is the number of stomatal openings in a picture divided by the total number of stomatal openings.

## Data Availability

The RNA-seq data has been deposited in NCBI Gene Expression Omnibus (GEO) under accession code GSE136047.

## Additional Points

*Preprint Explanation*. A preprint of this manuscript was released online [45].

## Supplementary Materials

Supplementary Materials
*Table S1*. The DEGs were detected in the comparisons of HR1 vs. HR0. *Table S2*. The DEGs were detected in the comparisons of HR3 vs. HR0. *Table S3*. The DEGs were detected in the comparisons of HR5 vs. HR0. *Table S4*. The DEGs were detected in the comparisons of HR7 vs. HR0. *Table S5*. Functional analysis of the DEGs from HR1 vs. HR0. *Table S6*. Functional analysis of the DEGs from HR3 vs. HR0. *Table S7*. Functional analysis of the DEGs from HR5 vs. HR0. *Table S8*. Functional analysis of the DEGs from HR7 vs. HR0.

## References

[1] Zhang X., Dong J., Deng F. (2019). The long non-coding RNA lncRNA973 is involved in cotton response to salt stress. *BMC Plant Biology*.

[2] Bashir K., Matsui A., Rasheed S., Seki M. (2019). Recent advances in the characterization of plant transcriptomes in response to drought, salinity, heat, and cold stress. *F1000Research*.

[3] Lesk C., Rowhani P., Ramankutty N. (2016). Influence of extreme weather disasters on global crop production. *Nature*.

[4] Wei T., Cherry T. L., Glomrod S., Zhang T. (2014). Climate change impacts on crop yield: evidence from China. *Science of the Total Environment*.

[5] Rendon J. L., Choudhry M. A. (2012). Th17 cells: critical mediators of host responses to burn injury and sepsis. *Journal of Leukocyte Biology*.

[6] Zhao C., Liu B., Piao S. (2017). Temperature increase reduces global yields of major crops in four independent estimates. *Proceedings of the National Academy of Sciences of the United States of America*.

[7] Rienth M., Torregrosa L., Luchaire N. (2014). Day and night heat stress trigger different transcriptomic responses in green and ripening grapevine (vitis vinifera) fruit. *BMC Plant Biology*.

[8] Zhang C., Bian Y., Hou S., Li X. (2018). Sugar transport played a more important role than sugar biosynthesis in fruit sugar accumulation during Chinese jujube domestication. *Planta*.

[9] Huang J., Zhang C., Zhao X. (2016). The jujube genome provides insights into genome evolution and the domestication of sweetness/acidity taste in fruit trees. *PLoS Genetics*.

[10] Gao Q. H., Wu C. S., Wang M. (2013). The jujube (Ziziphus jujuba Mill.) fruit: a review of current knowledge of fruit composition and health benefits. *Journal of Agricultural and Food Chemistry*.

[11] Liu M. J., Zhao J., Cai Q. L. (2014). The complex jujube genome provides insights into fruit tree biology. *Nature communications*.

[12] Li Z., Chen Y., Fang G., Li Y. (2017). Multivariate assessment and attribution of droughts in Central Asia. *Scientific Reports*.

[13] Wang C., He W., Kang L., Yu S., Wu A., Wu W. (2019). Two-dimensional fruit quality factors and soil nutrients reveals more favorable topographic plantation of Xinjiang jujubes in China. *PLoS One*.

[14] Zhuang Q., Wu S., Feng X., Niu Y. (2020). Analysis and prediction of vegetation dynamics under the background of climate change in Xinjiang, China. *PeerJ*.

[15] Li C., Liu H., Huang F. (2014). Effect of temperature on the occurrence and distribution of Colorado potato beetle (Coleoptera: Chrysomelidae) in China. *Environmental Entomology*.

[16] Jin J., Yang L., Fan D., Liu X., Hao Q. (2020). Comparative transcriptome analysis uncovers different heat stress responses in heat-resistant and heat-sensitive jujube cultivars. *PLoS One*.

[17] Ohama N., Sato H., Shinozaki K., Yamaguchi-Shinozaki K. (2017). Transcriptional regulatory network of plant heat stress response. *Trends in Plant Science*.

[18] Kim D., Langmead B., Salzberg S. L. (2015). HISAT: a fast spliced aligner with low memory requirements. *Nature Methods*.

[19] Robinson M. D., McCarthy D. J., Smyth G. K. (2010). edgeR: a Bioconductor package for differential expression analysis of digital gene expression data. *Bioinformatics*.

[20] da Huang W., Sherman B. T., Lempicki R. A. (2009). Systematic and integrative analysis of large gene lists using DAVID bioinformatics resources. *Nature Protocols*.

[21] Livak K. J., Schmittgen T. D. (2001). Analysis of relative gene expression data using real-time quantitative PCR and the 2(-Delta Delta C(T)) method. *Methods*.

[22] Richter K., Haslbeck M., Buchner J. (2010). The heat shock response: life on the verge of death. *Molecular Cell*.

[23] Sun A. Z., Guo F. Q. (2016). Chloroplast retrograde regulation of heat stress responses in plants. *Frontiers in plant science*.

[24] Driedonks N., Rieu I., Vriezen W. H. (2016). Breeding for plant heat tolerance at vegetative and reproductive stages. *Plant reproduction*.

[25] Bita C. E., Gerats T. (2013). Plant tolerance to high temperature in a changing environment: scientific fundamentals and production of heat stress-tolerant crops. *Frontiers in plant science*.

[26] Gonzalez-Schain N., Dreni L., Lawas L. M. (2016). Genome-wide transcriptome analysis during anthesis reveals new insights into the molecular basis of heat stress responses in tolerant and sensitive rice varieties. *Plant & Cell Physiology*.

[27] Shi J., Yan B., Lou X., Ma H., Ruan S. (2017). Comparative transcriptome analysis reveals the transcriptional alterations in heat-resistant and heat-sensitive sweet maize (Zea mays L.) varieties under heat stress. *BMC Plant Biology*.

[28] Qin D., Wu H., Peng H. (2008). Heat stress-responsive transcriptome analysis in heat susceptible and tolerant wheat (Triticum aestivum L.) by using Wheat Genome Array. *BMC Genomics*.

[29] Mathur S., Agrawal D., Jajoo A. (2014). Photosynthesis: response to high temperature stress. *Journal of Photochemistry and Photobiology B: Biology*.

[30] Jumrani K., Bhatia V. S., Pandey G. P. (2017). Impact of elevated temperatures on specific leaf weight, stomatal density, photosynthesis and chlorophyll fluorescence in soybean. *Photosynthesis Research*.

[31] Caine R. S., Yin X., Sloan J. (2019). Rice with reduced stomatal density conserves water and has improved drought tolerance under future climate conditions. *The New Phytologist*.

[32] Hetherington A. M., Woodward F. I. (2003). The role of stomata in sensing and driving environmental change. *Nature*.

[33] Putarjunan A., Torii K. U. (2020). Heat shocking the Jedi master: HSP90’s role in regulating stomatal cell fate. *Molecular Plant*.

[34] Samakovli D., Ticha T., Samaj J. (2020). HSP90 chaperones regulate stomatal differentiation under normal and heat stress conditions. *Plant Signaling & Behavior*.

[35] Han S. K., Qi X., Sugihara K. (2018). MUTE directly orchestrates cell-state switch and the single symmetric division to create stomata. *Developmental cell*.

[36] Shu Y., Tao Y., Wang S. (2015). GmSBH1, a homeobox transcription factor gene, relates to growth and development and involves in response to high temperature and humidity stress in soybean. *Plant Cell Reports*.

[37] Zhao P. X., Miao Z. Q., Zhang J., Chen S. Y., Liu Q. Q., Xiang C. B. (2020). Arabidopsis MADS-box factor AGL16 negatively regulates drought resistance via stomatal density and stomatal movement. *Journal of Experimental Botany*.

[38] Mangelsen E., Kilian J., Harter K., Jansson C., Wanke D., Sundberg E. (2011). Transcriptome analysis of high-temperature stress in developing barley caryopses: early stress responses and effects on storage compound biosynthesis. *Molecular Plant*.

[39] Qian Y., Ren Q., Zhang J., Chen L. (2019). Transcriptomic analysis of the maize (Zea mays L.) inbred line B73 response to heat stress at the seedling stage. *Gene*.

[40] Zhao Y., Hu F., Zhang X. (2019). Comparative transcriptome analysis reveals important roles of nonadditive genes in maize hybrid An’nong 591 under heat stress. *BMC Plant Biology*.

[41] Chen S., Li H. (2016). Heat stress regulates the expression of genes at transcriptional and post-transcriptional levels, revealed by RNA-seq in Brachypodium distachyon. *Frontiers in Plant Science*.

[42] Jiang J., Liu X., Liu C., Liu G., Li S., Wang L. (2017). Integrating omics and alternative splicing reveals insights into grape response to high temperature. *Plant Physiology*.

[43] Yadav R., Lone S. A., Gaikwad K., Singh N. K., Padaria J. C. (2018). Transcriptome sequence analysis and mining of SSRs in Jhar Ber (Ziziphus nummularia (Burm.f.) Wight & Arn) under drought stress. *Sci Rep*.

[44] Guo M., Li S., Tian S., Wang B., Zhao X. (2017). Transcriptome analysis of genes involved in defense against alkaline stress in roots of wild jujube (Ziziphus acidojujuba). *PLoS One*.

[45] Yang L., Jin J., Fan D. Y., Hao Q., Niu J. X. (2021). *Transcriptome analysis of a jujube (Ziziphus Jujuba Mill.) cultivar response to heat stress*.

